# An exploratory study of dog park visits as a risk factor for exposure to drug-resistant extra-intestinal pathogenic *E. coli* (ExPEC)

**DOI:** 10.1186/s13104-015-1103-2

**Published:** 2015-04-10

**Authors:** Lubna N Ahmed, Lance B Price, Jay P Graham

**Affiliations:** Department of Environmental & Occupational Health, Milken Institute School of Public Health, George Washington University, 950 New Hampshire Ave. NW, Washington, DC, 20052 USA

**Keywords:** Dogs, Extraintestinal pathogenic *E. coli*, Antibiotic resistance

## Abstract

**Background:**

Extraintestinal pathogenic *E. coli* (ExPEC) are common causative agents of urinary tract infections in humans. Dogs have been found to harbor ExPEC. This study tested stool samples from dogs (n = 16), the shoes of dog park visitors (n = 16) and the shoes of controls (n = 16) for ExPEC. Phenotypic resistance of isolates was characterized.

**Findings:**

ExPEC were present in one-third of the dog stool samples, 9% of the samples from the shoes of dog park visitors and 6% of control samples. Half of the ExPEC isolates were multi-drug resistant.

**Discussion:**

The findings suggest that dogs may be an important source of antibiotic resistant ExPEC.

**Electronic supplementary material:**

The online version of this article (doi:10.1186/s13104-015-1103-2) contains supplementary material, which is available to authorized users.

## Background

Extraintestinal pathogenic *Escherichia coli* (ExPEC) are one of the leading causes of urinary tract infections [1]. In 2011, the American Veterinary Medical Association estimated that there were more than 70 million pet dogs in the United States – roughly one dog for every five people [2]. Dogs are a known reservoir of ExPEC [3]. Furthermore, veterinarians use a broad spectrum of antibiotics for pet health [4]. The use of antibiotics can lead to the presence of antibiotic resistant bacteria in pet feces, which have the potential to contaminate public spaces, such as parks, and can or may contribute to community-acquired infectious diseases [5,6]. Human and canine isolates of multi-drug resistant ExPEC clonal groups have been found to be closely related, suggesting possible cross-host species transfer [7,8].

In this exploratory study, we assessed the prevalence of ExPEC in stool specimens taken from two urban dog parks in Washington, D.C, USA, as well as the prevalence of ExPEC on the soles of shoes of dog park visitors and controls. These locations may be important sites for transmission of zoonotic pathogens and drug resistant bacteria [9].

In the fall of 2012, we collected freshly voided dog stool samples (n = 16) and subsequently swabbed the shoe soles of corresponding dog care takers at two dog parks (n = 16), as well as the shoe soles of individuals walking on sidewalks (i.e. controls) approximately 200 meters from the dog parks (n = 16). All samples were collected aseptically and were processed for non-typed *E. coli* within four hours of collection.

Stool samples were directly streaked onto Difco™ Violet Red Bile Agar with 4-methylumbelliferyl-bD-glucuronide (VRBA-MUG) and incubated for 24 hours at 37°C as previously described [10]. Swab samples were placed in Thermo Scientific™ Remel™ MacConkey Broth and incubated for two hours at 37°C, followed by 22 hours at 44°C [11]. After incubation a 10 ul loopful of this processed enrichment broth was also streaked on to VRBA-MUG and incubated for 24 hours. Isolates exhibiting similar fluorescence to the *E. coli* control (ATCC 25922), were streaked to HardyCHROM™ ECC and incubated 20 to 24 hours at 37°C.

*E. coli* isolates were placed in a 20% glycerol stock and frozen at −20°C. The antibiotic susceptibility of each *E. coli* isolate was determined by the disk diffusion method according to CLSI guidelines and included 12 different antibiotics (see Additional file 1). Quantitative PCR was used to confirm the identity of putative *E. coli* isolates (see Additional file 1). *E. coli* isolates were tested for six ExPEC hallmark genes: *papA, sfaE, kpsMII, papC, iutA, and afaC. E. coli* isolates positive for two or more of the six hallmark virulence genes were classified as ExPEC (see Additional file 1).

This study was determined to be exempt from human subject protection by The George Washington University Institutional Review Board given that the study did not involve collecting personal information on human subjects and because the proposed uses and disclosures of protected health information involved no more than minimal risk to the privacy of individuals (45 CFR 46 164.512). Written informed consent was obtained from all participants prior to sample collection.

## Findings

A total of 16 dog stool specimens, 16 dog park visitor shoe swabs and 16 control shoe swabs were taken. Fourteen dog stool samples (88%) and 14 swabs from the soles of shoes of dog park visitors (88%) were positive for *E. coli*. Eleven of the control shoe swabs (69%) were positive for *E. coli*. Antibiotic resistance was low among all the isolates. Of the 15 unique *E. coli* isolates from dog stool samples, two isolates (13%) were resistant to two or more of the 12 antibiotics tested. Four *E. coli* isolates from the shoes of dog park visitors (17%) were resistant to two or more antibiotics – three of these were resistant to four to seven antibiotics. Only one *E. coli* isolate from controls (6%) was resistant to two or more antibiotics (Table 1). *E. coli* from the shoes of dog park visitors displayed a higher level of resistance to all antibiotic classes in comparison to isolates collected from control shoe swabs. Resistance to *ampicillin* was an exception – an average of 20% of all *E. coli* isolates were resistant. *Tetracycline* resistance was common among *E. coli* isolates from dog stools (13%) and the shoes from dog park visitors (17%). None of the isolates from the shoes of controls were resistant to *tetracycline*. Resistance to both *ciprofloxacin* and *nalidixic acid* was observed in one *E. coli* isolate from dog stool and one *E. coli* isolate from the shoes of a dog park visitor (these stool and shoe swab isolates did not belong to a corresponding pair).

**Table 1:** **Characterization of** ***E. coli*** **isolates based on antibiotic resistance and ExPEC status**

**Antibiotic class and antibiotic name**	**Dog stool sample** ***E. coli*** **isolates n = 15**	**Park visitor shoe swab** ***E. coli*** **isolates n = 23**	**Control shoe swab** ***E. coli*** **isolates n = 16**
	**(% resistant)**	**(% resistant)**	**(% resistant)**
**Penicillins**			
*Ampicillin*	3 (20.0%)	5 (21.7%)	3 (18.8%)
*Ampicillin Sulbactam*	0 (0.0%)	2 (8.7%)	1 (6.3%)
**Cephems**			
*Cefazolin*	0 (0.0%)	3 (13.0%)	1 (6.3%)
*Cefoxitin*	0 (0.0%)	2 (8.7%)	1 (6.3%)
*Ceftriaxone*	0 (0.0%)	2 (8.7%)	1 (6.3%)
**Quinolones**			
*Ciprofloxacin*	1 (6.7%)	1 (4.3%)	0 (0.0%)
*Nalidixic acide*	1 (6.7%)	1 (4.3%)	0 (0.0%)
**Tetracycline**			
*Tetracycline*	2 (13.3%)	4 (17.4%)	0 (0.0%)
**Folate Pathway Inhibitors**			
*Trimethoprim-sulfamethoxozole*	0 (0.0%)	2 (8.7%)	0 (0.0%)
**Aminoglycosides**			
*Amikacin*	0 (0.0%)	0 (0.0%)	0 (0.0%)
*Gentamicin*	1 (6.7%)	0 (0.0%)	0 (0.0%)
**Carbapenem**			
*Imipenem*	0 (0.0%)	0 (0.0%)	0 (0.0%)
**Multi-Drug Resistant**			
**(≥2 antibiotics)**	2 (13.3%)	4 (17.4%)	1 (6.3%)
**Extraintestinal pathogenic** ***E. coli*** ^***1***^	**Number (%) ExPEC**	**Number (%) ExPEC**	**Number (%) ExPEC**
	5 (33%)	2 (9%)	1 (6%)
**Multi-Drug Resistant ExPEC** ^**1**^	**Number (%)**	**Number (%)**	**Number (%)**
**(≥2 antibiotics)**	**Multi-Drug Resistant**	**Multi-Drug Resistant**	**Multi-Drug Resistant**
	2 (13%)	2 (9%)	0 (0%)

^1^Percent ExPEC and percent multi-drug resistant is calculated from total *E. coli* isolates.

Eight of the total 54 *E. coli* isolates were identified as ExPEC and displayed multi-drug resistance – one isolate was resistant to two drugs, five of the isolates were resistant to three drugs, and two of the isolates were resistant to four drugs. Five out of the 15 *E. coli* isolates from dog stool samples were ExPEC (33%); two out of the 23 *E. coli* isolates from the shoes of dog park visitors were ExPEC (9%), and one out of the 16 isolates from controls was identified as ExPEC (Table 1). Among the ExPEC hallmark genes that were identified, *kpsMII* was the most common, with a total of 11 *E. coli* isolates across all three sample groups. The *papA* gene was found among dog fecal isolates and park visitor shoe swab isolates (seven *E. coli* isolates (18%). Also commonly found among all three sampling groups was *SfaE* with a total of seven *E. coli* isolates.

## Discussion

The results of this study suggest that dogs may be important contributors to the spread of antibiotic resistant ExPEC. Additionally, visits to dog parks, where caretakers of pets often take dogs to defecate, may be important sites for the transmission of antibiotic resistant ExPEC. Individuals returning from such locations may unknowingly spread bacteria via shoes or other points of contact into their households. Bringing attention to these sites, as potential points of exposure is important, as dissemination of drug resistant bacteria from these sources can put large populations at risk of becoming ill. Urinary tract infections for example, are the most common infectious diseases contracted by women in high-income countries [12]. Decreased effectiveness of antibiotics against drug-resistant bacterial strains can make such infections difficult or impossible to treat and lead to serious public health implications [13]. Further, exposure to ExPEC may disproportionately affect vulnerable populations such as children, elderly individuals, and those who have compromised immune systems [14]. Additional research would be useful for characterizing potentially important sites – such as dog parks – for exposure to antibiotic resistant ExPEC.

## Additional file

Additional file 1:
**Methods.**

## Abbreviations

ExPEC: Extra-intestinal Pathogenic *E. coli*
