# Supplementary material for: An exploratory study of dog park visits as a risk factor for exposure to drug-resistant extra-intestinal pathogenic E. coli (ExPEC)
Source: BMC Res Notes. 2015 Apr 10;8:137. doi: 10.1186/s13104-015-1103-2 (PMC4397871; doi:10.1186/s13104-015-1103-2)
Supplement: Additional file 1: — Methods. [file 13104_2015_1103_MOESM1_ESM.docx]

**METHODS**

*Species Confirmation*. For each putative *E. coli* isolate, a single colony was inoculated into an assigned well of a 96-well plate containing 75 μl of TE. DNA was then released from cell suspensions with a thermal cycler (Bio-Rad, Hercules, CA) using the following parameters: heated lid, 95°C; block temperature, 90°C for 15 min. Confirmed *E. coli* isolates (see Species Confirmation) were streaked to LB agar and incubated overnight at 37°C, then stored at -80°C in Brucella Broth with 20% glycerol. An in-house *uidA* quantitative PCR (qPCR) assay and a universal bacterial qPCR assay (BactQuant; 1) were used to confirm the identity of putative *E. coli* isolates. For each reaction, 2 μl of DNA was added into 8 μl of master mix, with the final reaction containing 1.8 μM of each forward and reverse *uidA* primer, 0.25 μM *uidA-*VIC probe, 0.90 μM of each forward and reverse Pan16S primer, 0.25 μM Pan16S-FAM probe, 1X QuantaPerfeCTa® Multiplex qPCR SuperMix w⁄ROX (Quanta Biosciences, Gaithersburg, MD) and molecular-grade water. All samples were run in triplicate and each experiment included a standard curve and no-template controls. The 7900HT Real-Time PCR System (Applied Biosystems, Carlsbad, CA) was used to run the reactions with the following conditions: 3 min at 50°C for UNG treatment, 10 min at 95°C for Taq activation, 15 s at 95°C for denaturation and one min at 60°C for annealing and extension x 40 cycles.

*Susceptibility testing*. The antibiotic susceptibility of each *E. coli* isolate was determined by the disk diffusion method according to CLSI guidelines and included the following 12 antibiotics: amikacin (AMK), ampicillin (AMP), ampicillin-sulbactam (SAM), cefazolin (CFZ), cefoxitin (FOX), ceftriaxone (CRO), ciprofloxacin (CIP), gentamicin (GEN), imipenem (IPM), nalidixic acid (NAL), tetracycline (TET), and trimethoprim-sulfamethoxazole (SXT). Isolates were classified as “susceptible”, “intermediate”, or “resistant” in accordance with breakpoint guidelines from the 2011 CLSI M100^9^ Tables 2A through 2J for *E. coli*; for statistical analyses “intermediate” and “resistant” isolates were grouped together.

The 12 individual antibiotics used for susceptibility testing fell into seven different antibiotic classes: aminoglycosides (amikacin and gentamicin), carbapenems (imipenems), cephalosporins (cefazolin, cefoxitin, and ceftriaxone), folate pathway inhibitors (trimethoprim-sulfamethoxazole), penicillins and penicillin combinations (ampicillin and ampicillin-sulbactam), quinolones (nalidixic acid and ciprofloxacin), and tetracyclines (tetracycline). Isolates resistant to two or more classes of antibiotics were classified as multi-drug resistant (MDR).

*Extraintestinal Pathogenic E. coli (ExPEC) testing*. *E. coli* isolates were tested for six ExPEC hallmark genes: *papA, sfaE, kpsMII, papC, iutA, and afaC*. For the first triplex qPCR, 2 μl of DNA was added into 8 μl of master mix, with the final reaction containing 0.50 μM of each forward and reverse *afaC* primer, 0.13 μM *afaC-*FAM probe, 0.10 μM of each forward and reverse *papC* primer, 0.10 μM *papC*-HEX probe, and 0.50 μM of each forward and reverse *iutA* primer, 0.13 μM *iutA-*RED probe, 1X QuantaPerfeCTa® Multiplex qPCR SuperMix w⁄ROX (Quanta Biosciences, Gaithersburg, MD) and molecular-grade water. For the second triplex reaction, 2μl of DNA was added into 8 μl of master mix, with the final reaction containing 0.10 μM of each forward and reverse *papA* primer, 0.13 μM *papA-*FAM probe, 0.50 μM of each forward and reverse *kpsMII* primer, 0.13 μM *kpsMII*-HEX probe, and 0.30 μM of each forward and reverse *sfaE* primer, 0.13 μM *sfaE-*RED probe, 1X QuantaPerfeCTa® Multiplex qPCR SuperMix w⁄ROX (Quanta Biosciences, Gaithersburg, MD) and molecular-grade water. The Roche Light Cycler 480 (Roche, Pleasanton, CA) was used to run the reactions with the following conditions: hot start at 95 °C for three min; amplification for 45 cycles of 95 °C for 15 s (ramp rate 4.8 °C/s), 55°C for one min (acquisition single, ramp rate 2.5 °C/s); followed by a cooling step at 40 °C for 10 s (ramp rate of 2.5 °C/s). Fluorescence signal was obtained by using Mutli Hydrolysis Probe setting for FAM (nm), HEX (nm), and RED (nm). *E. coli* isolates positive for two or more of the six hallmark virulence genes were classified as ExPEC.
